# Rapeseed and Raspberry Seed Cakes as Inexpensive Raw Materials in the Production of Activated Carbon by Physical Activation: Effect of Activation Conditions on Textural and Phenol Adsorption Characteristics

**DOI:** 10.3390/ma9070565

**Published:** 2016-07-12

**Authors:** Koen Smets, Mats De Jong, Iwona Lupul, Grazyna Gryglewicz, Sonja Schreurs, Robert Carleer, Jan Yperman

**Affiliations:** 1 Research Group of Applied and Analytical Chemistry, CMK, Hasselt University, Agoralaan Gebouw D, Diepenbeek 3590, Belgium; koen_smets@hotmail.be (K.S.); mats.dejong@student.uhasselt.be (M.D.J.); robert.carleer@uhasselt.be (R.C.); 2 Division of Polymer and Carbonaceous Materials, Faculty of Chemistry, Wroclaw University of Technology, ul. Gdañska 7/9, Wroclaw 50-344, Poland; iwona.lupul@pwr.edu.pl (I.L.); grazyna.gryglewicz@pwr.edu.pl (G.G.); 3 NuTeC, CMK, Hasselt University, Agoralaan Gebouw H, Diepenbeek 3590, Belgium; sonja.schreurs@uhasselt.be

**Keywords:** activated carbon, textural characterization, phenol adsorption

## Abstract

The production of activated carbons (ACs) from rapeseed cake and raspberry seed cake using slow pyrolysis followed by physical activation of the obtained solid residues is the topic of this study. The effect of activation temperature (850, 900 and 950 °C), activation time (30, 60, 90 and 120 min) and agent (steam and CO_2_) on the textural characteristics of the ACs is investigated by N_2_ adsorption. In general, higher activation temperatures and longer activation times increase the BET specific surface area and the porosity of the ACs, regardless of the activation agent or raw material. Steam is more reactive than CO_2_ in terms of pore development, especially in the case of raspberry seed cake. The performance of the ACs in liquid adsorption is evaluated by batch phenol adsorption tests. Experimental data are best fitted by the Freundlich isotherm model. Based on total yield, textural characteristics and phenol adsorption, steam activation at 900 °C for 90 min and CO_2_ activation at 900 °C for 120 min are found as the best activation conditions. Raspberry seed cake turns out to be a better raw material than rapeseed cake. Moreover, AC from raspberry seed cake produced by steam activation at 900 °C for 90 min performs as well as commercial AC (Norit GAC 1240) in phenol adsorption. The adsorption kinetics of the selected ACs are best fitted by the pseudo-second-order model.

## 1. Introduction

Activated carbons (ACs) are widely used as adsorbents in the purification of waste streams. Phenols, acid dyes, pesticides and heavy metals are common pollutants in liquid waste streams, while the removal of VOCs, NO_x_ and SO_x_ is frequently the objective of air pollution control. Consequently, ACs are used in various industries, including food and beverage processing, pharmaceutical, chemical, petroleum, mining, nuclear and automobile industries [1].

AC production generally involves a carbonization step (in inert atmosphere) followed by physical or chemical activation. Carbonization (pyrolysis) is usually performed between 400 and 850 °C in order to remove considerable amounts of tar and volatile matter from the raw material, resulting in a solid residue with a relatively high carbon content and a preliminary porosity [2,3]. This raw porosity, however, is usually not developed enough for most AC applications, and additional activation to create new pores and/or to unclog and to widen existing ones is required.

In physical activation, carbon atoms are removed (gasified) from the solid residue by using mild oxidizing agents, such as steam and CO_2_, at temperatures between 800 and 1000 °C. Different mechanisms are reported in the literature for both activation agents. Steam is believed to attack the active sites at the pore center and on the pore walls simultaneously, while CO_2_ primarily reacts with active sites located at the pore center (creating micropores) and only attacks the pore walls when the activation time becomes longer (broadening of micropores). As a result, CO_2_ requires more time to develop new and to widen existing micropores. Therefore, steam is considered as more reactive than CO_2_ under analogous conditions [2,4]. The higher reactivity of steam is assigned to its more advantageous kinetics. Hence, H_2_O as steam has a smaller molecular size than CO_2_ and, therefore, can diffuse faster through the pore network and gain easier access to (existing) micropores [4,5]. As a result, steam generally produces ACs with broader pore size distributions and larger surface areas, whereas CO_2_ is more selective towards the creation of micropores.

Regular AC production is expensive due to the use of non-renewable and/or relatively costly raw materials (e.g., coal, lignite, peat or wood) [1,6]. Recently, there has been a growing interest in using inexpensive materials, such as agricultural by-products and wastes, as raw the material in AC production [2,7,8,9]. Therefore, rapeseed cake and raspberry seed cake (two agricultural waste cakes) are investigated as raw material for AC production in this study for two reasons. Firstly, to the knowledge of the authors, these agricultural waste cakes have not been investigated for their opportunities as raw materials in AC production so far. Secondly, the authors already published the valorization of both waste cakes by pyrolysis with the main focus on the production and characterization of the pyrolysis liquid [10,11,12]. As a result, this study complements the previous work by investigating the valorization of the solid fraction obtained by slow pyrolysis.

First, slow pyrolysis up to 450 °C was used to produce the solid residues, which were subsequently converted to ACs using physical activation. The effect of various activation conditions (temperature, time and activation agent) on the yield and characteristics of the ACs was studied. The ACs were characterized by FTIR, ATR-FTIR and SEM and texturally by N_2_ adsorption, and their performance in liquid adsorption was evaluated by batch phenol adsorption experiments. Phenol was chosen as the target compound due to its occurrence in many wastewaters and its detrimental effects on human health and aquatic life [13,14].

## 2. Material and Methods

### 2.1. Raw Material

Two agricultural waste cakes were evaluated as raw material for the production of AC. Rapeseed cake (moisture: 1.5 wt %; volatile matter: 75.5 wt %; fixed carbon: 18.1 wt %; and ash: 4.9 wt %) was obtained from cold-pressing of “double low” winter rapeseed (*Brassica napus* L.) in the production of high quality vegetable oil. Raspberry seed cake (moisture: 1.5 wt %; volatile matter: 68.5 wt %; fixed carbon: 27.4 wt %; and ash: 2.6 wt %) was obtained after supercritical CO_2_-extraction of raspberry seeds (*Rubus idaeus* L.) in the production of high value antioxidants and vegetable oil. For an in-depth characterization of both agricultural waste cakes, the reader is referred to the previous work of the authors [10,11,12]. Prior to carbonization, both raw materials were ground to a particle size smaller than 2 mm and dried in an oven at 110 °C.

### 2.2. AC Production

Firstly, the raw material (about 140 g) was heated (10 °C/min) to 450 °C and carbonized at this temperature for 1 h in a lab-scale reactor set-up, resulting in a solid residue. In contrast to previous work of the authors [10], the reactor set-up was operated without sand as the heat transfer medium in order to obtain a solid residue free from sand and clean for further activation. The solid residue, obtained after carbonization, was cooled down under flowing N_2_, sieved (particle size: 125–700 µm), washed with hot distilled water and dried at 110 °C. Secondly, the solid residue (about 7 g) was converted to AC in a horizontal tubular quartz reactor using physical activation. Hereby, it was placed as a fixed bed between quartz wool plugs and heated (10 °C/min) from room temperature to activation temperature under flowing N_2_ (70 mL/min). Then, the activation agent (steam: 0.1 mL/min of liquid water in 70 mL/min N_2_ or CO_2_: 70 mL/min) was applied for a certain activation time. The effect of activation temperature was tested at 850, 900 and 950 °C for a fixed time of 30 min, while the effect of activation time was studied by four experiments (30, 60, 90 and 120 min) at 900 °C. The obtained ACs are designated R-X-T-t, where R indicates the raw material (RSC and RBC for rapeseed cake and raspberry seed cake, respectively), X is the activation agent (S and C for steam and CO_2_, respectively), T the activation temperature and t the activation time. Finally, the sample was cooled down under flowing N_2_, washed with hot distilled water, dried at 110 °C and stored in a desiccator. Prior to characterization, an additional HCl washing step was performed on the ACs produced from RSC due to their very high ash content. Therefore, these ACs were boiled under reflux with HCl (0.1 M) for 1 h, washed with distilled water to remove remaining HCl and dried at 110 °C. Since ACs from RBC had a much lower ash content, no HCl washing step was performed on these samples.

### 2.3. AC Characterization

N_2_ adsorption isotherms were measured with a NOVA 2200 gas sorption analyzer (Quantachrome Instruments) at 77 K in the relative pressure (p/p_0_) range from 0.008 to 0.98. Prior to analysis, ACs were outgassed overnight at 300 °C. The specific surface area (S_BET_) was calculated using the BET method [15,16]. The micropore volume (V_DR_) was determined by applying the Dubinin–Radushkevich equation in the p/p_0_ range from 0.008 to 0.05 [17]. The total pore volume (V_t_) was obtained from the volume of N_2_ adsorbed at p/p_0_ = 0.96, while the mesopore volume (V_me_) was estimated by subtracting V_DR_ from V_t_ [1,4]. The average micropore size (L_0_) was calculated using the Stoeckli equation [18]. Elemental composition (CHNS) of the ACs was determined using a FlashEA 1112 Elemental Analyzer (Thermo Electron Corporation). Oxygen was calculated by the difference. Ash content was determined as the residual weight after combustion at 850 °C by TGA [11]. The FTIR spectra were recorded with a Brüker Vertex 70 FTIR spectrometer. The sample was mixed with KBr in the ratio of 1:250. Typically, 32 scans/min were taken with a resolution of 4 cm^−1^ and a background correction was performed. The functional groups of the ACs were analyzed using attenuated total reflectance Fourier transform infrared (ATR-FTIR) spectroscopy. Spectra were recorded with a Brüker Vertex 70 FTIR spectrometer equipped with a HYPERION 1000 microscope and an MCT (mercury cadmium telluride) detector. Typically, 32 scans/min were taken with a resolution of 4 cm^−1^, and a background correction was performed.

The surface morphology of the ACs was examined by scanning electron microscopy (SEM) using an FEI Quanta 200 FEG-SEM apparatus at IMO (Instituut voor Materiaalonderzoek, Diepenbeek, Belgium). Imaging was performed in high vacuum mode at an accelerating voltage of 15.0 kV using an ET (Everhart-Thornley) detector.

### 2.4. Phenol Adsorption Study

#### 2.4.1. Batch Phenol Adsorption

Batch phenol adsorption tests were carried out in 100 mL glass Erlenmeyer flasks using 25 mg of AC and 25 mL of phenol solution. Phenol solutions (initial concentrations between 5 and 400 mg/L) were prepared in distilled water without pH adjustment (initial pH about 6). After being placed in a shaking water bath at 25 °C for 48 h, the ACs were removed from the phenol solution by filtration (Whatmann 40). The remaining phenol concentration was determined by the direct photometric method [19]. In this method, phenol reacts with 4-aminoantipyrine to form a stable reddish-brown-colored antipyrine dye. The amount of color, measured at 550 nm using a Pharmacia Biotech Ultrospec 2000 UV-VIS spectrophotometer is related to the amount of phenol. Six phenol solutions were used for calibration, and distilled water, subjected to a similar procedure as the unknown samples, was used as the blank. Samples were diluted, if necessary, to fit the calibration range (1.0–10.0 mg/L). The precision of phenol determination is between 3% and 5% within the concentration range of 1.0–10.0 mg/L.

#### 2.4.2. Effect of the Initial pH of the Phenol Solution

The effect of the initial pH of the solution on the phenol removal was studied for pH values between 4 and 13 using phenol solutions with an initial concentration of 200 mg/L. The initial pH of the phenol solutions (about 6) was adjusted by adding small amounts of HCl (0.1 M) or NaOH (0.1 M). The pH-values were measured with a Hamilton Polilyte Lab electrode.

#### 2.4.3. Adsorption Isotherm Models

Langmuir and Freundlich models were used to fit the adsorption isotherms and to evaluate the isotherm parameters, since they are widely the most accepted models for single-solute systems [20]. The Langmuir model can be written as [21]:

(1) $$q_{e}=\frac{q_{m}{\;K}_{L}{\;C}_{e}}{\left({1+K}_{L}\;C_{e}\right)}$$

where q_e_ (mg/g) is the amount of solute adsorbed per mass unit of adsorbent at equilibrium, C_e_ (mg/L) the equilibrium concentration, q_m_ (mg/g) the Langmuir monolayer adsorption capacity and K_L_ (L/mg) the Langmuir constant, which is related to the free energy of adsorption. This model is valid for monolayer adsorption on a homogeneous surface containing a finite number of sites that have an equal adsorption energy and that are equally available for adsorption. It assumes uniform adsorption on the surface and no transmigration of adsorbate on the plane of the surface [7,20]. The Freundlich model is an empirical equation based on sorption on a heterogeneous surface or surfaces supporting sites of varied affinities and with interaction between the adsorbate molecules [20]. It is assumed that stronger binding sites are occupied first and that the binding strength decreases with increasing degree of surface coverage [7]. This model is described by Equation (2) [22]:

(2) $$q_{e}=K_{F}\;C_{e}^{1/n_{F}}$$

where K_F_ ((mg/g)(L/mg)^1/n^_F_) is indicative of the relative adsorption capacity of the adsorbent as it represents the quantity of adsorbate at an equilibrium concentration of 1 mg/L, and 1/n_F_ (dimensionless number) is a measurement of the adsorption intensity or surface heterogeneity [7,23]. Hence, a 1/n_F_ value closer to zero represents a more heterogeneous surface [20]. Since the Freundlich model does not impose any requirement that the coverage must approach a constant value corresponding to a complete monolayer at high equilibrium concentrations of adsorbate, the failure of this model might be expected at high concentrations [24]. In this study, the model parameters were calculated by applying non-linear regression using the method of least squares (OriginLab Origin 7.0 software).

#### 2.4.4. Kinetic Study

The kinetics of phenol adsorption was evaluated using the pseudo-first-order model, suggested by Lagergren [25], and the pseudo-second-order model, introduced by Blanchard et al. [26]. The models are described by Equations (3) and (4), respectively.

(3) $$q_{t}=q_{e}\left(1\;-\text{ exp}\left(-k_{1}t\right)\right)$$

(4) $$q_{t}=\frac{k_{2}q_{e}^{2}t}{\left({1+\text{k}}_{2}q_{e}t\right)}$$

with q_t_ and q_e_ (mg/g) the amount of phenol adsorbed per mass unit of AC after a contact time t (min) and at equilibrium, respectively, k_1_ (1/min) the pseudo-first-order rate constant and k_2_ (g/mg min) the pseudo-second-order rate constant. The model parameters (q_e_ and k_1_ or k_2_) were calculated by non-linear regression according to the method of least squares. Kinetic studies were performed by loading the AC (about 25 mg) into nine 100 mL glass Erlenmeyer flasks containing a phenol solution (25 mL) with an initial concentration of 100 mg/L. The samples were placed in a shaking water bath (25 °C) for a certain period of time. After removal of the AC by filtration, the remaining phenol concentration in the aqueous phase was quantified by UV-VIS.

## 3. Results and Discussion

### 3.1. AC Production 

Carbonization of rapeseed cake and raspberry seed cake at 450 °C for 1 h resulted in respectively 27.3 wt % and 34.9 wt % of solid residue. Each solid residue was further converted to AC using physical activation. The burn-off, ash content and total AC yield are shown in Table 1 for the various activation conditions.

The burn-off, representing the amount of solid residue removed during physical activation, generally increased for higher activation temperatures and longer activation times, regardless of the activation agent or raw material. Comparison of the activation agents revealed that steam was more reactive than CO_2_ under corresponding conditions for both raw materials.

The ACs produced from RSC had a very high ash content (25.4–57.7 wt %), which is undesirable for the adsorption of phenol. Therefore, these ACs were submitted to an additional HCl washing step. Although complete ash removal was not achieved, this additional step reduced the ash content considerably (7.1–21.3 wt %). The ACs produced from RBC had a relatively low ash content (5.7–13.0 wt %) and therefore did not require additional HCl washing.

The total AC yield is an important economic parameter representing the amount of AC obtained from 100 g of raw material. For RSC, yields between 8.3 and 21.0 wt % were obtained without HCl washing. The additional HCl washing step reduced the total AC yield (5.3–19.6 wt %), but resulted in ACs that performed (considerably) better in phenol adsorption than the corresponding untreated samples. Therefore, only HCl washed ACs were characterized in detail in the case of RSC. For ACs from RBC, the total yield varied between 12.8 and 29.3 wt %. These yields were quite satisfactory since values between 10 and 20 wt % are reported for an economically-viable AC production [27].

### 3.2. AC Characterization

#### 3.2.1. Nitrogen Adsorption Isotherms and Pore Size Distributions

**Figure 1:**
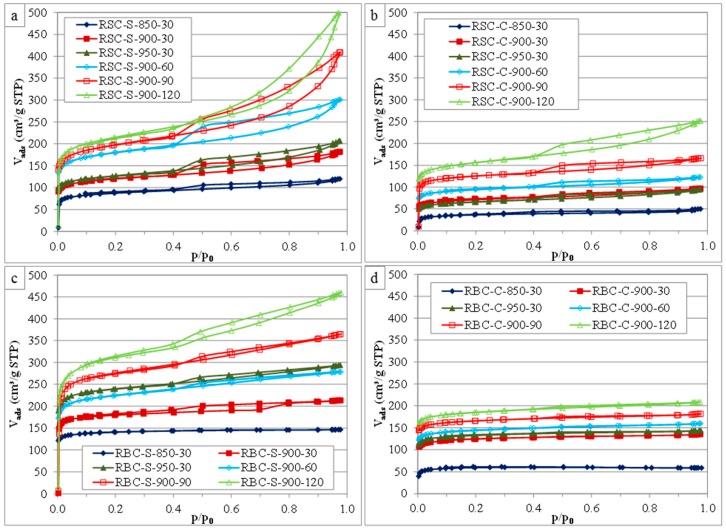
N_2_ adsorption isotherms of ACs produced from RSC by steam (**a**) and CO_2_ (**b**) activation and of ACs produced from RBC by steam (**c**) and CO_2_ (**d**) activation using various activation conditions. (STP = standard temperature and pressure).

The effects of the activation conditions on the porosity of the ACs were investigated by N_2_ adsorption at 77 K. An overview of the N_2_ adsorption isotherms is presented in Figure 1.

According to the IUPAC classification, the HCl-washed ACs produced from RSC (Figure 1a,b) showed type IV adsorption isotherms [28,29]. The steep initial part of the isotherm (p/p_0_ < 0.05) was assigned to micropore filling, indicating a considerable degree of microporosity (pore widths < 2 nm). The second part of the isotherm (p/p_0_ > 0.4) consisted of two separated branches, indicating hysteresis and associated capillary condensation in mesopores (pore widths: 2–50 nm). The hysteresis loops were classified as type H4, resembling those of microporous adsorbents with narrow slit-shaped pores [29]. These loops became more pronounced for more severe activation conditions. The ACs produced from RBC using steam activation (Figure 1c) had type IV adsorption isotherms with type H4 hysteresis loops, except for the sample activated at the lowest temperature (RBC-S-850-30). In contrast, the CO_2_ activated samples (Figure 1d) showed type I adsorption isotherms, except for the ones produced using longer activation times (RBC-C-900-90 and RBC-C-900-120). These results suggest that the activation agent had a considerable effect on the pore development when RBC was used as the raw material, while this was much less in the case for the HCl-washed ACs produced from RSC.

Several important textural characteristics of the ACs were inferred from the N_2_ adsorption isotherms (Table 2). Very low BET specific surface areas were found for the solid residues obtained by carbonization from RSC (about 0.5 m^2^/g) and RBC (about 20 m^2^/g). 

Higher activation temperatures increased the BET specific surface area and the pore volumes (V_t_, V_me_ and V_DR_) of the ACs, regardless of the activation agent or raw material; except for RSC-C-950-30, where a decrease in S_BET_, V_t_ and V_DR_ was observed between 900 and 950 °C, while V_me_ slightly increased. A partial destruction of micropores in favor of mesopores might explain this observation. In the case of RBC, temperatures above 850 °C were required for effective pore development by CO_2_ activation. The V_DR_/V_t_ ratio decreased as a function of activation temperature for all ACs, except for RBC-C-950-30. This indicated that the contribution of the micropore volume to the total pore volume decreased in favor of the mesopore volume, although both micro- and meso-pore volumes increased in absolute terms. An extension of the activation time from 30 to 120 min considerably increased the BET specific surface area and the porosity for all ACs, regardless of the activation agent or raw material. In the case of steam activation, the V_DR_/V_t_ ratio considerably decreased with longer activation times for both raw materials, while the average micropore diameter (L_0_) significantly increased. This indicated that micropores became wider and that mesopores had a larger contribution to the total pore volume for longer activation with steam. In contrast, CO_2_ activation affected the V_DR_/V_t_ ratio, but was much less pronounced. The V_DR_/V_t_ ratio first increased, remained constant (60–90 min) and then significantly decreased for ACs produced from rapeseed cake. The V_DR_/V_t_ ratio and the L_0_ value of the ACs produced from RBC by CO_2_ activation were hardly affected by an extension of the activation time (except for RBC-C-850-30). Steam was more effective than CO_2_ in terms of BET specific surface area and pore development under corresponding conditions. Moreover, this difference in reactivity was more pronounced for ACs produced from RBC, as already observed in the N_2_ adsorption isotherms (Figure 1). The ACs from RSC had considerably lower BET specific surface areas and micropore volumes than the commercial AC (Norit GAC 1240), while the mesopore volumes were more pronounced, especially for long activation times (Table 2). Steam activation at 900 °C for 90 min or 120 min turned out to be the best activation conditions in terms of BET specific surface area and pore development. In the case of ACs from RBC, sample RBC-S-900-90 showed a BET specific surface area and pore volume distribution that was very similar to the commercial AC. Moreover, even better textural characteristics were obtained for RBC-S-900-120. CO_2_ activation was less effective, but resulted in ACs that have the potential to be valorized in adsorption processes requiring microporous adsorbents.

The elemental composition of the solid residues and the ACs is presented in Table 3.

The ACs had a higher carbon content and a lower content of hydrogen and nitrogen than the corresponding solid residue: RSC-solid residues (SR) and RBC-SR. The oxygen content was hardly affected by activation in the case of RSC (except for RSC-C-900-30), while it was strongly reduced for ACs from RBC. Negative values were caused by experimental error due to the rather small sample size (about 3 mg) used in the elemental analysis. Sulfur contents below 1 wt % are usually desirable for ACs used as adsorbents [30]. This requirement was easily met for RBC, since only minor amounts of sulfur were detected in both solid residue and ACs. In contrast, ACs from RSC contained a considerable amount of sulfur, which seemed to decrease for longer activation times. Heteroatoms (such as O, N and S) are mostly part of the surface functional groups, and therefore, can play an important role in the surface chemistry and adsorption behavior of ACs [24].

#### 3.2.2. FTIR Analysis

**Figure 2:**
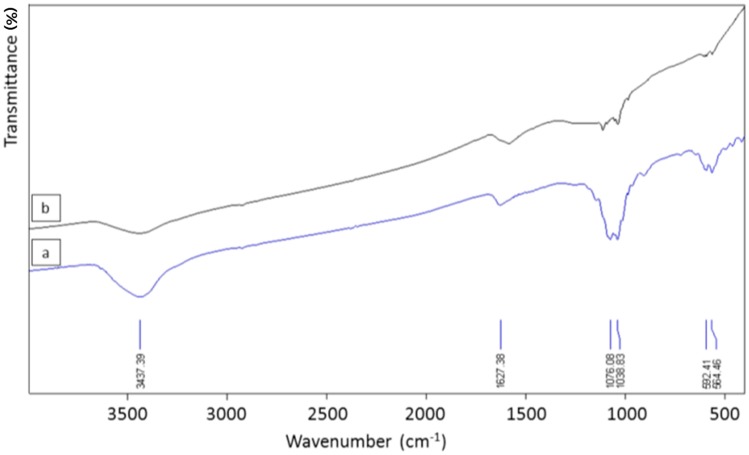
FTIR spectra of an AC (1 mg AC in 250 mg KBr) produced from rapeseed cake (S-900-120) before (**a**) and after (**b**) HCl washing.

Figure 2 shows the FTIR spectra of a representative sample of AC produced from rapeseed cake (steam activation at 900 °C for 120 min; S-900-120) before and after HCl washing. The intense absorption bands at 1076 cm^−1^ and 1039 cm^−1^ and at 592 cm^−1^ and 564 cm^−1^ were assigned to the symmetric and asymmetric stretch vibrations of P–O bonds and to bending vibrations of O–P–O, respectively. The intensity of these bands was considerably reduced by HCl washing, indicating that the compounds removed from the AC were mainly composed of salts of phosphates. This result was supported by ICP-AES analysis, which revealed P together with Ca, K and Mg as major elements in the HCl solution after HCl washing of the AC.

The ACs derived from raspberry seed cake had a much lower ash content (5.7–13.0 wt %) than those produced from rapeseed cake (Table 1). It was remarkable that the calculated ash contents were (considerably) higher than those determined by TGA. This suggested that part of ashes was “lost” during carbonization and activation. Because of their relatively low ash content, the ACs from raspberry seed cake were not treated by an additional HCl washing step.

#### 3.2.3. ATR-FTIR Functional Groups Characterization

**Figure 3:**
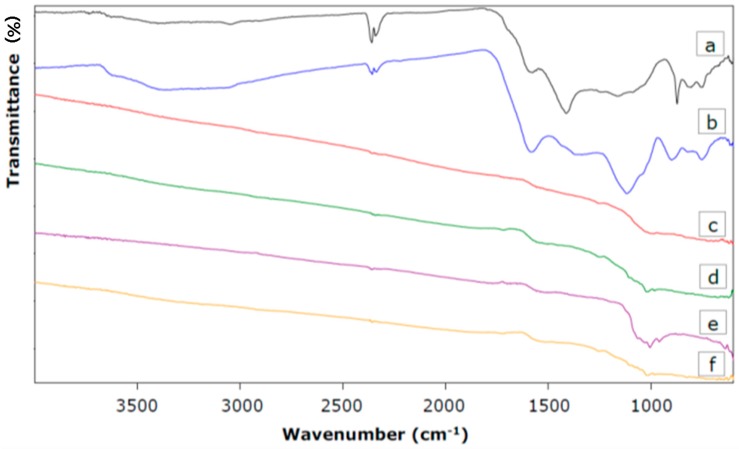
ATR-FTIR spectra of the solid residue from raspberry seed cake (**a**) and from rapeseed cake (**b**); of ACs produced from raspberry seed cake (**c**) and rapeseed cake (**d**) by CO_2_ activation (C-900-120); and ACs derived from raspberry seed cake (**e**) and rapeseed cake (**f**) by steam activation (S-900-90, HCl washed in the case of rapeseed cake).

In general, heteroatoms (such as oxygen and nitrogen) are important elements concerning the surface chemistry of ACs, since they are mostly part of organic functional groups at the edge of carbon crystallites. In this view, oxygen typically occurs in carboxyl, carbonyl, phenol, ether or lactone functionalities, while nitrogen is mainly found as a part of amines or nitro-groups [24]. Because surface functional groups can play an important role in adsorption processes, the solid residues and ACs were investigated by ATR-FTIR spectroscopy, as well. Figure 3 shows the spectra of the solid residues and of four representative ACs produced from both agricultural waste cakes.

Several broad overlapping bands were observed in the ATR-FTIR spectra of the solid residues (Figure 3a,b). Approximate band assignment suggested the presence of carboxyls, carbonyls, lactones, phenols, olefinic and aromatic structures. Hence, the spectral region of 1750–1550 cm^−1^ is associated with C=O stretching vibrations of carbonyls, carboxylic acids and lactones and with C=C bonds in olefinic and aromatic structures. The bands between 1460 and 1000 cm^−1^ could represent C–O and O–H bending vibrations [7]. However, the assignment of a specific wavenumber to a given functional group was not possible due to the overlap and shifts of the absorption bands of various functional groups [7].

The surface chemistry of activated carbons differed significantly from that of solid residues. Hence, almost no absorption bands were found in the case of the representative ACs (Figure 3c–f). This indicated that almost all surface functional groups were removed by the applied activation conditions.

#### 3.2.4. Surface Morphology of Activated Carbons 

**Figure 4:**
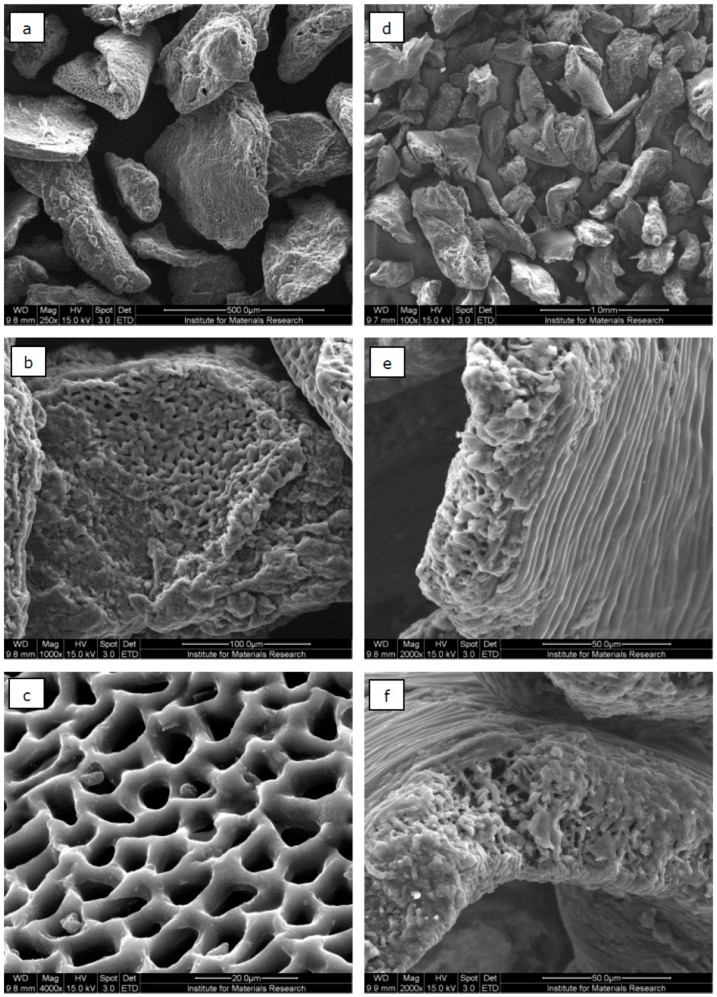
SEM images of ACs produced from rapeseed cake (C-900-60) with a magnification factor of 250 (**a**), 1000 (**b**) and 4000 (**c**); and ACs produced from raspberry seed cake (C-950-30) with a magnification factor of 100 (**d**), 2000 (**e**) and 4000 (**f**).

The ACs of both waste cakes were investigated by SEM. No systematic effect of the activation conditions (temperature, time and activation agent) on the morphology of the ACs could be observed from the SEM images. However, the morphology of the ACs did significantly depend on the precursor material. An overview of two representative samples is shown in Figure 4. 

In the case of rapeseed cake (Figure 4a–c), many AC particles had a sponge-like porous structure (Figure 4b), which consisted of long parallel, nearly cylindrically-shaped pores with quite uniform pore widths (Figure 4c). For the ACs derived from raspberry seed cake (Figure 4d–f), many particles showed a layered surface with porous edges (Figure 4e). A closer look at the edges (Figure 4f) revealed pores with non-uniform widths and quite irregular shapes.

### 3.3. Phenol Adsorption Study

#### 3.3.1. Effect of the Initial pH of the Phenol Solution

**Figure 5:**
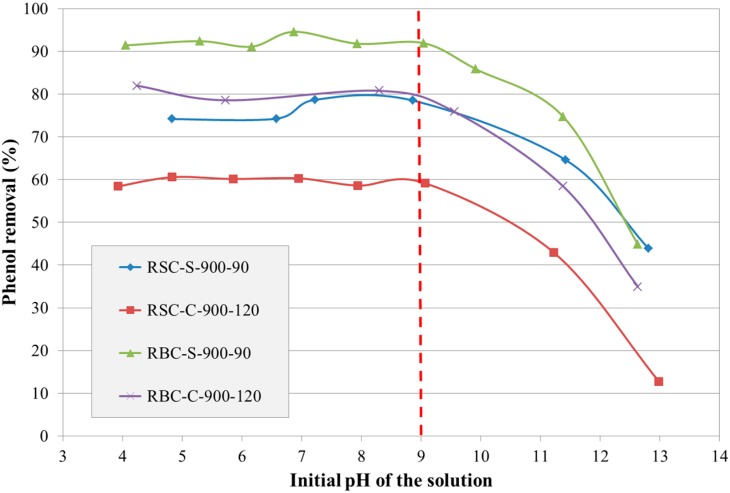
Effect of the initial pH of a solution on the phenol removal by selected ACs derived from RSC and RBC (C_0_ = 150 mg/L).

The initial pH of a solution is known to affect the adsorption of phenol by a combination of two factors: the acid-base behavior of phenol and the surface charge of the AC. Phenol is a weak acid with a pK_a_ of 9.89 (25 °C). As a result, the uncharged molecular form dominates at pH < 9.89, while the phenolate anion is the predominant form at pH > 9.89. On the other hand, surface functional groups might be either neutral or negatively charged at higher pH values [7]. Therefore, the effect of the solution’s initial pH on the phenol removal was investigated for selected ACs (Figure 5).

Figure 5 shows that phenol removal was rather independent of the solution’s initial pH for pH values below about nine, while it significantly decreased at more alkaline pH values for all selected ACs. Dispersive interactions between the aromatic ring of phenol and the basal planes with a high π-electron density of the AC were believed to be responsible for phenol removal at pH values below nine. On the other hand, electrostatic repulsion between the negative surface charge of the AC and the phenolate anions could explain the reduced phenol removal at more alkaline pH values. From this preliminary test, it is concluded that the solution’s initial pH does not affect the adsorption of phenol by the selected ACs considerably as long as it is below nine.

#### 3.3.2. Phenol Adsorption Isotherms

**Figure 6:**
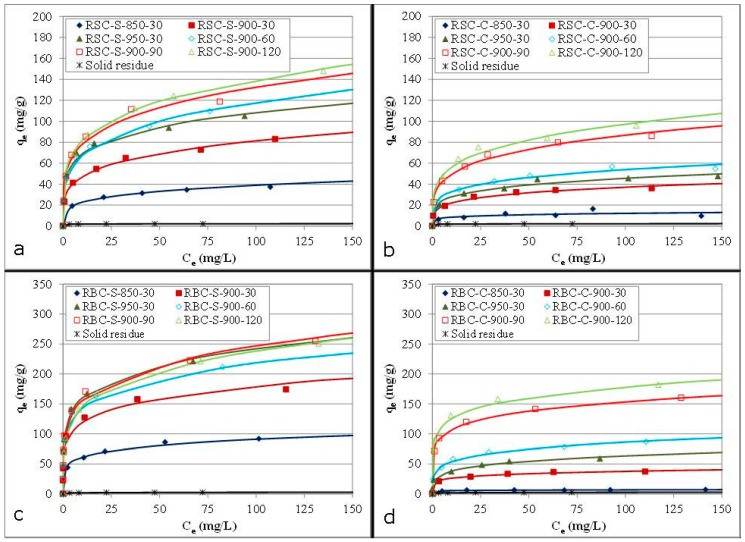
Phenol adsorption isotherms (dots: experimental data; lines: fit of the Freundlich model) of HCl-washed ACs produced from RSC by steam (**a**) and CO_2_ (**b**) activation and of ACs originating from RBC activated by steam (**c**) and CO_2_ (**d**).

The performance in liquid adsorption was evaluated for all ACs using batch phenol adsorption tests. Phenol adsorption isotherms are presented in Figure 6.

Without activation, the solid residues produced by the carbonization of RSC and RBC showed a very low phenol adsorption (both below 3 mg/g). In the case of the ACs, phenol adsorption was strongly improved by a higher activation temperature, regardless of the activation agent or raw material. Steam-activated samples performed much better than corresponding CO_2_-activated samples. Moreover, phenol adsorption was hardly improved by CO_2_ activation at 850 °C compared to the solid residue, suggesting that a temperature above 850 °C is required for CO_2_ activation to be effective. Phenol adsorption was also significantly improved by a longer activation time for all samples. Comparison of both raw materials revealed that ACs from RBC always performed better than corresponding HCl-washed ACs produced from RSC.

#### 3.3.3. Adsorption Models of Freundlich and Langmuir

The Freundlich and the Langmuir isotherm models were used to fit the experimental equilibrium data (C_e_, q_e_) for a better understanding of the adsorption mechanism (Table 4).

In general, the equilibrium data were best fitted by the Freundlich model, as indicated by the R^2^ values. Therefore, the ACs were believed to contain some heterogeneity on the surfaces and/or pores participating in phenol adsorption. The Freundlich constant (K_F_) increased with higher activation temperatures and longer activation times (except for RBC-S-900-120). The 1/n_F_ parameter had values less than unity for all samples, indicating that phenol adsorption was a favorable process [20]. For ACs from RBC, the 1/n_F_ parameter increased as a function of activation time for steam activation and as a function of activation temperature for CO_2_ activation, indicating a decrease of the surface heterogeneity in both cases [31]. In contrast, no systematic trend was found for this parameter for the HCl-washed ACs derived from RSC. The Langmuir model fitted the experimental data less well, but could still provide valuable information. In general, more severe activation conditions significantly increased the monolayer adsorption capacity (q_m_). The HCl-washed ACs from RSC performed not as well as commercial AC, regardless of the activation conditions. In contrast, ACs from RBC showed better adsorption parameters than the commercial AC for longer activation times and higher activation temperatures. Based on these results, the textural characteristics (Table 2) and the total AC yield (Table 1), steam activation at 900 °C for 90 min and CO_2_ activation at 900 °C for 120 min were selected as the best activation conditions for both raw materials.

#### 3.3.4. Kinetic Study

Kinetic parameters provide important information on the designing and modeling of the adsorption process [20]. Therefore, selected ACs were investigated by a kinetic study. Experimental data were modeled using the pseudo-first order and the pseudo-second order model (Table 5).

**Figure 7:**
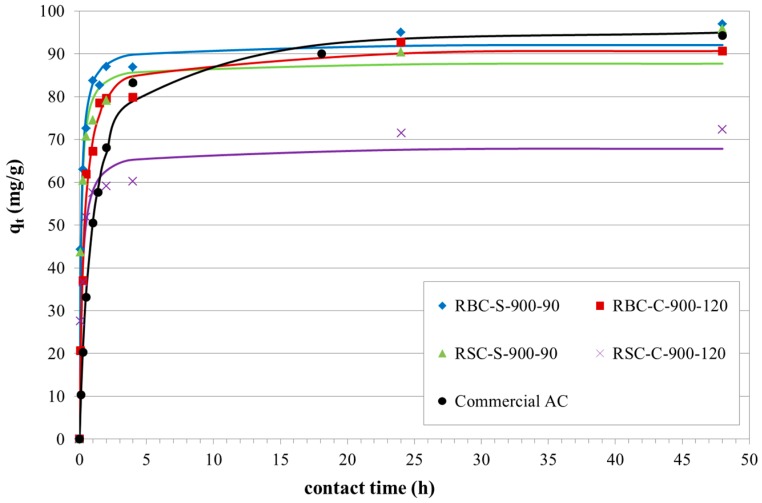
Phenol adsorption kinetic study (dots: experimental data; lines: fit of the PSO model) of the best performing ACs derived from RSC and RBC (C_0_ = 100 mg/L).

The adsorption process was best described by the pseudo-second order model for all best performing ACs, as indicated by the R^2^ values. The experimental data and the fit with this model are shown in Figure 7.

The results revealed that phenol adsorption was fast at the initial stage of adsorption and became slower near the equilibrium. Hence, a large number of vacant surface sites were available for adsorption during the initial stage, while for longer contact times, the remaining vacant surface sites were more difficult to occupy due to the small number of vacancies available, the less free space within the channels of the AC pores and the repulsive force between the phenol molecules on the AC and the ones in the bulk liquid phase [23]. The steam-activated samples reached equilibrium faster than the corresponding CO_2_-activated samples. The higher porosity of the former might explain this observation, since four consecutive mass transport steps can be distinguished for adsorption on a porous adsorbent [23,32]. Firstly, phenol molecules have to migrate through the bulk solution to the film surrounding the adsorbing particle. Then, the solute has to diffuse from the film to the surface of the particle (film diffusion) and subsequently from the particle surface into the interior site of the adsorbent (pore diffusion). Finally, phenol molecules are adsorbed on the active sites of the adsorbent [32,33].

**Figure 8:**
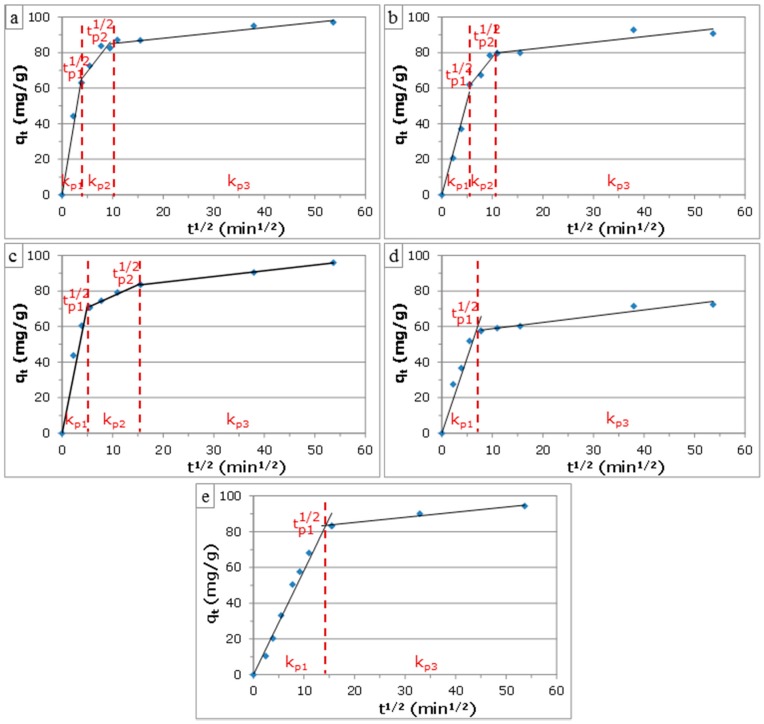
Plots of the intraparticle diffusion (IPD) model for phenol adsorption on selected ACs: (**a**) RBC-S-900-90; (**b**) RBC-C-900-120; (**c**) RSC-S-900-90; and (**d**) RSC-C-900-120; compared to commercial AC (**e**).

The intraparticle diffusion (IPD) model, presented by Weber and Morris [34], was applied on the kinetic data to study the diffusion mechanism. This empirically-found model assumes that the phenol uptake varies almost proportionally with the square root of time (t^1/2^), as shown by Equation (5):

q_t_ = k_pi_t^1/2^ + C_i_ (5)

where q_t_ (mg/g) is the amount of phenol adsorbed at time t (min), k_pi_ (mg/(g·min^1/2^)) is the IPD rate constant of stage i and C_i_ (mg/g) gives an idea about the boundary layer thickness, i.e., the larger C_i_, the greater the boundary layer effect [23,35]. If IPD occurs, the plot of q_t_ against t^1/2^ yields a straight line with k_pi_ as the slope and C_i_ as the intercept. If the plot for all points passes through the origin, then IPD is the only rate-limiting process. Otherwise, the plot may present multi-linearity, indicating that some other mechanism, together with IPD, is involved as well. In general, the rate of uptake might be limited by the size of adsorbate molecule, the adsorbate concentration, its affinity for the adsorbent, the diffusion coefficient of the adsorbate in the bulk phase, the pore size distribution of the AC and the degree of mixing [36]. Figure 8 shows the plots of the IPD model for selected ACs and the commercial AC. 

Up to three stages were distinguished. The first stage represented instantaneous adsorption or adsorption on the external surface of the AC (boundary layer diffusion). This stage was completed within 36 min and 60 min for ACs from RBC and RSC, respectively, while the commercial AC required about 205 min. This fast adsorption rate of the obtained ACs is an economical benefit in the case of continuous adsorption processes. The second stage was assigned to gradual adsorption with rate-limiting IPD. The third region was the final equilibrium stage, where IPD started to slow down due to extremely low adsorbate concentrations left in the solution [36,37]. This stage was only observed for ACs from RBC and the steam-activated sample from RSC (RSC-S-900-90), while it was absent for RSC-C-900-120. The multistage character of the IPD plots and the fact that the linear lines of the second and/or third stage did not pass through the origin indicated that IPD was not the only rate-limiting mechanism and that boundary layer diffusion also affected the adsorption to some extent [36,38]. The values of k_pi_, C_i_ and R^2^ were inferred from the IPD plots (Table 6).

For all ACs, the values of k_pi_ were found to decrease between Stages 1 and 3, while that of C_i_ increased. This indicated that the adsorption rate was slowed down and that the boundary layer effect became more pronounced, respectively, when contact time increased. ACs from RSC by steam activation had considerably higher k_p1_ and k_p2_ rate constants than the CO_2_-activated sample, while no k_p3_ was found for the latter. This might be explained by the higher porosity (both micropores and mesopores) of the steam-activated sample. In the case of ACs from RBC, the steam-activated sample had a considerably higher k_p1_ and a slightly higher k_p2_ rate constant than the CO_2_-activated sample, while almost no difference was found for k_p3_. The values of C_2_ and C_3_ were also considerably higher for the steam-activated than for the CO_2_-activated sample. No C_3_ value was found for RSC-C-900-120. This indicated that steam-activated samples had a faster adsorption on the external surface, a higher IPD and a greater boundary layer effect than CO_2_-activated samples.

## 4. Conclusions

The production of activated carbon (AC) from two agricultural waste cakes (i.e., RSC and RBC) is investigated using various activation conditions: three activation temperatures (850, 900 and 950 °C), four activation times (30, 60, 90 and 120 min) and two activation agents (steam and CO_2_). The activation conditions considerably affect the total yield and the textural characteristics of the ACs. Steam is more reactive than CO_2_, resulting in ACs with better textural characteristics. Better textural characteristics improve the adsorption of phenol. The phenol adsorption isotherms are best fitted by the Freundlich model. Based on total AC yield, textural characteristics and phenol adsorption, steam activation at 900 °C for 90 min and CO_2_ activation at 900 °C for 120 min are selected as the best activation conditions for both raw materials. Compared to commercial AC, the ACs from agricultural waste cake (especially RBC-S-900-90 and RBC-S-900-120) have beneficial potential for treating phenol-polluted wastewaters, as they adsorb faster. The kinetics of phenol adsorption is best described by the pseudo-first-order model. 

## Figures and Tables

**Table 1:** Burn-off, ash content and total yield of the activated carbons (ACs) produced from rapeseed cake (RSC) and raspberry seed cake (RBC) using various activation conditions.

Sample Code	Burn-off ^a^ (wt %)	Ash Content ^b^ (wt %)		Total AC Yield (wt %)	
		Before HCl Washing	After HCl Washing	Before HCl Washing	After HCl Washing
RSC-S-850-30	26.3	28.6	17.0	20.1	17.8
RSC-S-900-30	35.1	28.4	13.4	17.7	15.0
RSC-S-950-30	36.5	30.4	12.8	17.3	14.2
RSC-S-900-60	43.9	33.9	7.1	15.3	11.2
RSC-S-900-90	59.2	45.4	14.6	11.1	7.7
RSC-S-900-120	69.6	57.7	21.3	8.3	5.3
RSC-C-850-30	23.2	25.4	18.7	21.0	19.6
RSC-C-900-30	23.1	26.5	18.6	21.0	19.3
RSC-C-950-30	34.1	27.6	18.2	18.0	16.3
RSC-C-900-60	30.4	27.7	13.7	19.0	16.3
RSC-C-900-90	41.3	31.5	12.2	16.0	12.9
RSC-C-900-120	49.6	37.0	17.8	13.8	11.2
RBC-S-850-30	21.7	7.5	–	27.3	–
RBC-S-900-30	35.0	6.2	–	22.7	–
RBC-S-950-30	49.4	8.2	–	17.7	–
RBC-S-900-60	46.6	6.3	–	18.6	–
RBC-S-900-90	55.8	8.3	–	15.4	–
RBC-S-900-120	63.4	13.0	–	12.8	–
RBC-C-850-30	16.2	5.7	–	29.3	–
RBC-C-900-30	19.3	6.7	–	28.2	–
RBC-C-950-30	22.0	5.8	–	27.2	–
RBC-C-900-60	22.7	5.9	–	27.0	–
RBC-C-900-90	27.0	8.0	–	25.5	–
RBC-C-900-120	31.2	10.0	–	24.0	–

^a^ Burn-off (wt %) = (m_solid residue_ − m_activated carbon_)/m_solid residue_ × 100; ^b^ determined by TGA.

**Table 2:** Textural characteristics of ACs produced from RSC (after HCl washing) and RBC using various activation conditions. Commercial AC is included as a reference.

Sample	S_BET_ (m^2^/g)	V_t_ (cm^3^/g)	V_me_ (cm^3^/g)	V_DR_ (cm^3^/g)	V_DR_/V_t_	L_0_ (nm)
RSC-S-850-30	336	0.181	0.047	0.134	0.74	1.44
RSC-S-900-30	458	0.272	0.092	0.180	0.66	1.02
RSC-S-950-30	483	0.309	0.118	0.191	0.62	1.04
RSC-S-900-60	678	0.446	0.178	0.268	0.60	1.11
RSC-S-900-90	737	0.589	0.294	0.295	0.50	1.32
RSC-S-900-120	804	0.720	0.392	0.328	0.46	1.94
RSC-C-850-30	141	0.073	0.015	0.058	0.79	2.57
RSC-C-900-30	272	0.147	0.039	0.108	0.73	1.12
RSC-C-950-30	250	0.142	0.043	0.099	0.69	1.01
RSC-C-900-60	362	0.187	0.045	0.142	0.76	0.94
RSC-C-900-90	487	0.253	0.061	0.192	0.76	1.08
RSC-C-900-120	591	0.379	0.145	0.234	0.62	1.09
RBC-S-850-30	563	0.226	0.009	0.217	0.96	0.62
RBC-S-900-30	721	0.328	0.048	0.280	0.85	0.82
RBC-S-950-30	941	0.451	0.081	0.370	0.82	0.98
RBC-S-900-60	873	0.429	0.086	0.343	0.80	0.95
RBC-S-900-90	1081	0.559	0.117	0.442	0.79	2.02
RBC-S-900-120	1179	0.698	0.209	0.489	0.70	2.24
RBC-C-850-30	236	0.091	0.000	0.091	1.00	1.51
RBC-C-900-30	492	0.208	0.018	0.190	0.92	0.69
RBC-C-950-30	529	0.221	0.017	0.204	0.93	0.68
RBC-C-900-60	572	0.246	0.026	0.220	0.90	0.64
RBC-C-900-90	660	0.279	0.025	0.254	0.91	0.63
RBC-C-900-120	735	0.320	0.036	0.284	0.89	0.66
Commercial AC	1115	0.581	0.124	0.457	0.79	2.63

**Table 3:** Elemental analysis (wt %, air-dried basis) of the solid residues (SR) and the ACs produced from RSC (after HCl washing) and RBC using various activation conditions.

Sample	C	H	N	S	O	Ash
RSC-SR	64.5	2.5	5.8	0.3	5.6	21.3
RBC-SR	77.9	3.1	3.1	<0.1	12.1	3.8
RSC-S-850-30	68.8	1.6	3.9	1.2	7.5	17.0
RSC-S-900-30	77.0	1.3	3.5	1.0	3.8	13.4
RSC-S-950-30	77.1	1.1	3.0	1.4	4.6	12.8
RSC-S-900-60	82.6	1.2	3.4	0.5	5.2	7.1
RSC-S-900-90	75.8	1.1	2.7	0.6	5.2	14.6
RSC-S-900-120	69.8	1.2	1.5	0.4	5.8	21.3
RSC-C-850-30	69.4	1.2	4.9	1.4	4.4	18.7
RSC-C-900-30	73.9	1.2	4.6	1.5	0.2	18.6
RSC-C-950-30	72.9	1.1	4.2	1.3	2.3	18.2
RSC-C-900-60	73.3	1.0	4.6	0.2	7.2	13.7
RSC-C-900-90	77.1	1.1	4.7	0.3	4.6	12.2
RSC-C-900-120	72.1	1.6	3.9	0.6	4.0	17.8
RBC-S-850-30	89.1	1.1	2.1	<0.1	0.2	7.5
RBC-S-900-30	89.1	0.9	1.5	<0.1	2.4	6.2
RBC-S-950-30	88.7	0.9	1.5	<0.1	0.7	8.2
RBC-S-900-60	88.9	0.8	1.3	<0.1	2.7	6.3
RBC-S-900-90	88.7	0.9	1.3	<0.1	0.9	8.3
RBC-S-900-120	86.7	1.0	1.1	<0.1	–	13.0
RBC-C-850-30	87.7	0.8	2.1	<0.1	3.6	5.7
RBC-C-900-30	89.5	0.9	2.2	<0.1	0.7	6.7
RBC-C-950-30	88.9	0.8	2.2	<0.1	2.3	5.8
RBC-C-900-60	88.6	0.8	2.3	<0.1	2.4	5.9
RBC-C-900-90	88.3	0.8	2.2	<0.1	0.7	8.0
RBC-C-900-120	88.6	0.8	2.2	<0.1	–	10.0

Apparently negative values due to experimental error (small sample size); standard deviations (3 independent measurements) are within 0.3–0.7 for C, 0.1–0.3 for H, S and O and 0.1–0.2 for N.

**Table 4:** Phenol adsorption isotherm models: model parameters and correlation coefficients of solid residues and ACs produced from RSC (after HCl washing) and RBC.

Sample	Freundlich Model			Langmuir Model		
	K_F_ ((mg/g)(L/mg)^1/nF^)	1/n_F_	R^2^	q_m_ (mg/g)	K_L_ (L/mg)	R^2^
RSC-S-850-30	13	0.24	0.991	41	0.13	0.944
RSC-S-900-30	27	0.24	0.996	78	0.23	0.944
RSC-S-950-30	43	0.20	0.987	96	0.60	0.952
RSC-S-900-60	38	0.25	0.993	104	0.30	0.955
RSC-S-900-90	45	0.23	0.980	116	0.39	0.966
RSC-S-900-120	47	0.24	0.999	139	0.19	0.955
RSC-C-850-30	6	0.16	0.765	12	0.25	0.785
RSC-C-900-30	13	0.23	0.989	36	0.20	0.954
RSC-C-950-30	17	0.22	0.982	48	0.14	0.973
RSC-C-900-60	20	0.22	0.985	56	0.16	0.970
RSC-C-900-90	28	0.24	0.991	84	0.20	0.960
RSC-C-900-120	32	0.24	0.986	93	0.20	0.970
RBC-S-850-30	40	0.18	0.994	94	0.25	0.960
RBC-S-900-30	79	0.18	0.978	153	2.05	0.931
RBC-S-950-30	103	0.18	0.995	184	2.88	0.898
RBC-S-900-60	91	0.19	0.985	195	1.69	0.914
RBC-S-900-90	96	0.21	0.988	216	1.04	0.891
RBC-S-900-120	88	0.22	0.999	223	0.40	0.911
RBC-C-850-30	4	0.11	0.980	7	0.41	0.985
RBC-C-900-30	18	0.16	0.996	38	0.30	0.974
RBC-C-950-30	23	0.22	0.990	53	0.94	0.933
RBC-C-900-60	33	0.21	0.993	84	0.25	0.936
RBC-C-900-90	75	0.16	0.998	155	0.51	0.951
RBC-C-900-120	88	0.15	0.994	174	0.93	0.946
RSC-SR	1.7	0.06	0.990	2.2	1.23	0.998
RBC-SR	1.4	0.16	0.983	2.6	0.41	0.982
Commercial AC	56	0.28	0.983	159	0.33	0.953

**Table 5:** Parameters of the pseudo-first-order (PFO) and the pseudo-second-order (PSO) model of selected ACs produced from RSC (after HCl washing) and RBC, compared to those of commercial AC.

Sample	PFO Model			PSO Model		
	q_e_ (mg/g)	k_1_ (1/min)	R^2^	q_e_ (mg/g)	k_2_ (g/mg·min)	R^2^
RSC-S-900-90	83	0.11	0.927	88	0.0018	0.978
RSC-C-900-120	64	0.064	0.930	68	0.0014	0.973
RBC-S-900-90	87	0.098	0.947	92	0.0017	0.989
RBC-C-900-120	84	0.039	0.968	91	0.00060	0.990
Commercial AC	90	0.013	0.991	97	0.00019	0.997

**Table 6:** IPD model constants and correlation coefficients for the adsorption of phenol on selected ACs derived from RSC and RBC, compared to commercial AC.

Sample	kp_i_ (mg/g·min^1/2^)			C_i_ (mg/g)			Corr. Coeff.			t^1/2^_p1_	t^1/2^_p2_
	kp_1_	kp_2_	kp_3_	C_1_	C_2_	C_3_	R^2^_1_	R^2^	R^2^_3_	(min^1/2^)	(min^1/2^)
RSC-S-900-90	14.38	1.28	0.32	0	64.31	78.46	0.924	0.985	0.999	4.91	14.74
RSC-C-900-120	8.49	0.35	–	0	55.28	–	0.911	0.943	–	7.79	–
RBC-S-900-90	17.14	3.68	0.30	0	50.99	82.09	0.978	0.875	0.920	3.79	9.20
RBC-C-900-120	10.60	3.53	0.31	0	42.10	76.42	0.980	0.935	0.807	5.95	10.66
Commercial AC	5.83	0.29	–	0	79.32	–	0.978	0.969	–	14.32	–

Corr. Coeff.: correlation coefficient.
